# The Human Population: Accepting Earth’s Limitations

**DOI:** 10.1289/ehp.112-a979b

**Published:** 2004-12

**Authors:** Steven Earl Salmony

**Affiliations:** Disability Determination Services, Raleigh, North Carolina, E-mail: sesalmony@aol.com

I thank Fowler and Hobbs for their letter (2004) and their research (2003). The view that a complexity of factors impacts human population growth certainly makes sense, and they have correctly pointed out that scientifically organized efforts to deal with human problems must take account of manifold interconnected events. Although it is necessary to recognize and acknowledge the complexities inherent in cultural life and the natural world, it is equally important that a dizzying array of variables not blind us to certain scientific facts of biophysical reality. Humankind is bound by such predominant facts because the workings of the world exist independently of human wishes and beliefs.

With this in mind, I thank Hopfenberg for his article (2003) in which he provided an elegant model that accounts for the salient factors governing the dynamics of global human population numbers. According to his findings, the size of the human population is determined primarily by food availability.

The realization that these two points of view differ—that there is complexity and simplicity in the world we inhabit—does not necessarily mean that one is correct and the other incorrect. To the contrary, it could be that each point of view is valid based on the scope of observation.

It may be somehow not quite right to agree with the entire idea of Hobbs and Fowler (2004) that “human population size is beyond human capacity to list, comprehend, and synthesize” without noticing that the same can be said regarding any observable phenomenon. Reality is likely just as complex as Hobbs and Fowler described; but it is also clear from the research of Hopfenberg (2003) and Hopfenberg and Pimentel (2001) that the dynamics of human population growth is no longer preternatural but knowable, and that the population dynamics of *Homo sapiens* is not essentially different from the population dynamics of other species in both the complexity and the simplicity of the governing elements.

A comprehensive and objective approach to human problems and human potentiality must acknowledge that humankind is a part of the biophysical world, not apart from it. Although Hobbs and Fowler (2004) are correct to note the control human culture exercises in “value systems, economics, politics and religion” in taking account of what is real, human and environmental health could be increasingly at risk because humanity denies scientific facts over which living beings may not have control.

In light of the different sets of data presented by Fowler and Hobbs (2003) and by Hopfenberg (2003), perhaps it is a misnomer for Hobbs and Fowler (2004) to uniformly describe the many, complicated ways humanity is changing the natural world as an “unprecedented success.” Are particulate and solid-waste pollution or the conversion of biomass into human mass with resulting bio-diversity loss examples of success? Perhaps the economic success of the prevailing culture is not sustainable and cannot be maintained much longer. Unbridled economic globalization, unrestricted increases in human consumption of resources, and growing absolute human population numbers are negatively affecting Earth by degrading its fitness as a habitat for humans and other species.

A point in human history may have been reached when the scale and rate of growth of economic expansion, the consumption of natural resources, and the increasing human population can be seen as patently unsustainable. Understanding the causes of and limits to humanity’s impact in the world is a necessary step toward changing human production, consumption, and population trends. Regardless of how long a culture prizes growth and chooses to leave it unchecked, surely it is not too late to accept limits to growth of the human economy, human consumption, and human numbers worldwide by altering human behavior accordingly.
